# Preparation and Characterization of New and Low-Cost Ceramic Flat Membranes Based on Zeolite-Clay for the Removal of Indigo Blue Dye Molecules

**DOI:** 10.3390/membranes13110865

**Published:** 2023-10-31

**Authors:** Yassine Khmiri, Afef Attia, Hajer Aloulou, Lasâad Dammak, Lassaad Baklouti, Raja Ben Amar

**Affiliations:** 1 Research Unit “Advanced Technologies for Environment and Smart Cities”, Faculty of Science of Sfax, University of Sfax, Sfax 3038, Tunisia; yasssinlac@gmail.com (Y.K.);; 2 Department of Chemical, Preparatory Institute for Engineering Studies of Gabes, University of Gabes, Gabes 6029, Tunisia; 3 CNRS, ICMPE, UMR 7182, Université Paris-Est Créteil, 2 rue Henri Dunant, 94320 Thiais, France; 4 Department of Chemistry, College of Sciences and Arts at Ar Rass, Qassim University, Ar Rass 51921, Saudi Arabia; blkoty@qu.edu.sa; 5 Laboratory of Applied Chemistry and Natural Substances Resources and Environment, Faculty of Sciences, University of Carthage, Zarzouna, Bizerta 7021, Tunisia

**Keywords:** smectite, zeolite, composite membrane, microfiltration, dye removal

## Abstract

Composite flat membranes were prepared using a dry uniaxial pressing process. The effect of the sintering temperature (850–950 °C) and smectite proportion (10–50 wt.%) on membrane properties, such as microstructure, mechanical strength, water permeability, and treatment performances, was explored. It was observed that increasing the sintering temperature and adding higher amounts of smectite increased the mechanical strength and shrinkage. Therefore, 850 °C was chosen as the optimum sintering temperature because the composite membranes had a very low shrinkage that did not exceed 5% with high mechanical strength, above 23 MPa. The study of smectite addition (10–50 wt.%) showed that the pore size and water permeability were significantly reduced from 0.98 to 0.75 µm and from 623 to 371 L·h^−1^·m^−2^·bar^−1^, respectively. Furthermore, the application of the used membranes in the treatment of indigo blue (IB) solutions exhibited an almost total turbidity removal. While the removal of color and COD decreased from 95% to 76%, respectively, they decreased from 95% to 52% when the amount of smectite increased. To verify the treated water’s low toxicity, a germination test was performed. It has been shown that the total germination of linseed grains irrigated by M_S10-Z90_ membrane permeate was identical to that irrigated with distilled water. Finally, based on its promising properties, its excellent separation efficiency, and its low energy consumption, the M_S10-Z90_ (10 wt.% smectite and 90 wt.% zeolite) sintered at 850 °C could be recommended for the treatment of colored industrial wastewater.

## 1. Introduction

Industrial progress has resulted in a large volume of chemical waste contaminating water and the environment [1]. Wastewater coming from different industrial activities such as textile, paper, and electroplating, among others, contains a diverse variety of organic contaminants [2]. The release of dye-containing wastewater into the environment is a major concern. Due to their complex nature and stability, many of these dyes are difficult to remove [3,4]. Among these dyes, indigo blue (IB), which is an organic dye, is usually used in textile industries. The many industrial applications of IB all show that this organic dye is a source of water pollution and can cause serious environmental problems. Therefore, the removal of dyes from wastewater has become a serious priority due to their toxicity [5,6]. Several conventional methods have been applied to eliminate organic dye-contaminated effluents from wastewater, such as electrochemical technology, biological treatment, membrane separation, and advanced oxidation processes [7,8]. However, these technologies suffer from a variety of restrictions, such as high operational costs. Consequently, developing low-cost solutions to successfully remove dye pollutants from effluents has become a challenge [9].

Physical treatment, such as membrane technology, has recently captured the attention and interest of several researchers. In addition, replacing organic membranes with ceramic membranes is progressively being used in many sectors due to their appealing features [10] such as improved chemical and thermal stability, good mechanical strength, long lifetime, and low environmental effects [11]. According to the literature, major attempts have been taken to fabricate novel ceramic membranes utilizing less expensive materials rather than typical industrial oxides (Al_2_O_3_, TiO_2_, and ZrO_2_, among others), which are costly and require a high sintering temperature currently superior to 1200 °C [12]. From a technical perspective, several authors have investigated the development of low-cost microfiltration ceramic membranes exploiting local and natural resources. Natural pozzolan was employed to produce a flat microfiltration membrane, which was applied to treat textile effluent [13]. The elaborated membranes were tested to treat the washing water of jeans. The findings revealed that they removed 99% of turbidity. Another natural material was employed to prepare low-cost ceramic membranes such as a clay/phosphate mixture that was used to synthesize flat microfiltration membranes for seawater desalination and industrial wastewater treatment [14]. This ceramic membrane exhibited high turbidity removal efficiency in all tested effluent feeds (99.80%), seawater (99.62%), and synthetic solutions (99.86%). Samhari et al. [15] investigated the fabrication of a flat microfiltration membrane from Moroccan kaolinite for the treatment of raw seawater. The filtration results showed a turbidity removal of 73% and 99%, respectively, for the raw seawater and agrifood model effluent.

The pressing technique is the most suitable for preparing flat membranes due to its simplified and quick shaping process compared to other usual preparation techniques: extrusion and calendaring [16]. For extrusion, it is very essential to add some chemical additives such as a binder and a plasticizer to raw materials to obtain a homogenous plastic paste [17]. Nevertheless, these organic additives can cause many disadvantages, such as an increase in membrane price, air pollution by decomposition, and/or evaporation during the sintering process, etc. [18]. The dry pressing method has received increasing interest due to the possibility of fabricating dense membranes without defects by applying high pressure often superior to 954 MPa [19]. Furthermore, this approach could affect both the microstructure and the physicochemical properties of the membrane. Lorente-Ayza et al. [20] demonstrated that a pressed flat membrane support presented higher porosity and a larger pore size than an extruded tubular membrane support using the same powders based on clay, chamotte, and calcite. From an energetic point of view, the dry pressing technique does not require water, which consumes energy during the drying step. Further, the compression of raw materials leads to an increase in the contact between grains, which need a lower sintering temperature compared to non-compact materials [21]. For all these reasons, the dry pressing technique could be considered to be an efficient method for low-cost membrane preparation.

In this context, the current work describes the development of flat microfiltration membranes by mixing natural zeolite and smectite via a dry pressing method. The raw materials are characterized by their beneficial properties and reduced cost.

The main benefits of this approach were that the raw materials used in this study were abundant and cost-effective. The mixture of zeolite with smectite provides new interesting properties in terms of membrane structure and morphology, which play a crucial role during the membrane filtration process. In addition to a decrease in the sintering temperature, it allows also a reduction of the shrinkage and swelling phenomena usually observed with clay material when it is used separately. It is noteworthy that with pure zeolite material, it was not easy to prepare a membrane with a good structure without cracks formation by the pressing method. In addition, the resulting membrane from a mixture of zeolite/smectite showed excellent properties during filtration related to color removal despite being in the ultrafiltration domain.

The effect of the sintering temperature and membrane composition on resulting properties such as porosity, microstructure, mechanical strength, and permeability was investigated. Furthermore, the efficiency of the composite microfiltration membranes was evaluated during the treatment of the colored solution. The reuse of treated water by the optimized membrane in agriculture remains a challenge for the treatment of membrane processes. Based on the literature, seed germination and seedling growth were at their maximum when using a treated colored solution and could be better than the control solution [22,23,24]. Hussain et al. [22] investigated the effect of sugar mill effluent on the growth and antioxidative potential of maize seedlings, and they reported that the treated effluent increased the growth parameters in the maize seedlings. Kathirvel et al. [23] studied the effect of dye effluent on the growth, yield, and biochemical attributes of Bengal gram. They considered that at 20% concentration of dye effluent, the plant showed maximum germination. Furthermore, Mahawar and Akhtar [24] demonstrated that the reduction in the percentage of seed germination at higher concentrations of dye in industrial effluent may be due to the higher amount of solids present in these effluents, which causes changes in the osmotic relationship of the seed and water. The results proved that diluted effluent could be effective for soybean crop irrigation. Consequently, in this study, water toxicity was determined through a germination test on linseeds. The model dye chosen is indigo blue, which is one of the most used dyes in the textile industry, especially in the jeans industry. We also chose it as part of the development of a global, inexpensive, and economically reliable solution for the treatment of effluents from an industrial partner, SITEX Company of Ksar Hellel–Tunisia.

## 2. Experiment

### 2.1. Membrane Materials

The raw materials used to prepare the flat composite ceramic membranes were smectite (S) and zeolite (Z). The first constituent was extracted from Jbel Stah (located about 20 km west of the city of Gafsa in the center-west of Tunisia). The main constituents of smectite are silica (49.9 wt.%) and alumina (19.77 wt.%) (Table 1). The chemical composition analysis showed that the Turkish zeolite is essentially composed of 73.3 wt.% silica and 11.75 wt.% alumina, besides the low amounts of other oxides such as K_2_O, TiO_2_, CaO, and Fe_2_O_3_ (Table 1). The characteristics of raw powders were detailed in previous papers [25,26].

Both raw materials are inexpensive and still very abundant in many countries, particularly in the developing world. As a result, obtained membranes can be produced at a low cost.

### 2.2. Elaboration of Flat Composite Membranes

To study the effect of mixed zeolite and smectite on the characteristics of ceramic membranes, three different membrane compositions were considered using raw samples prepared under the same conditions (Table 2). The choice of these compositions with a maximum smectite content less than or equal to 50 wt.% was the result of preliminary tests showing that beyond this content, shrinkage and swelling become too significant to ensure good morphology of the membrane.

**Figure 1:**
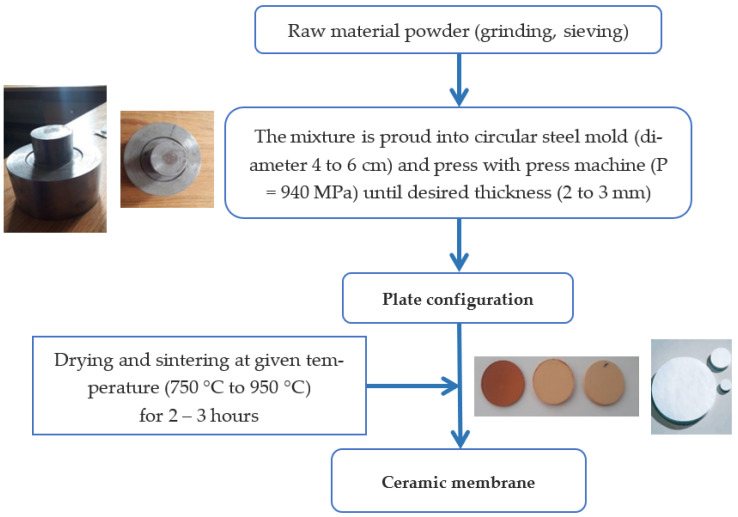
Schematic illustration of the ceramic membrane preparation by the pressing method.

Firstly, the materials were crushed and then sieved to a fine powder with a mesh size of 100 µm. Secondly, the homogeneous mixture was uni-axially compacted under 940 MPa in a cylindrical mold forming flat disks, which were then dried in an oven at 100 °C. Finally, the sintering was carried out in a programmed furnace at various temperatures. Figure 1 gives an illustration of this procedure.

The thermal analysis data were used to develop the customized firing procedure. Two steps have been specified, the first for the elimination of organic additives at 300 °C/2 h and the second for the sintering at various temperatures over 3 h [26]. The temperature–time schedule is mostly determined by porosity, surface quality, and the mechanical behavior of the final membrane. The prepared flat circular membranes had a diameter of 50 mm and were 3 mm thick.

### 2.3. Ceramic Membrane Characterization

The composite circular membranes prepared from different percentages of smectite and zeolite, M_S10-Z90_, M_S30-Z70_, and M_S50-Z50_, were sintered at different temperatures ranging from 750 to 950 °C. The best sintering temperature was chosen based on the visual aspect, the shrinkage rate, and the mechanical tests. Then, the estimated pore size, the chemical resistance, water permeability, and the performances in the IB solutions treatment were determined for the optimal composition of the composite membrane.

The shrinkage rate (*S* (%)) was determined from the measurement of the membrane diameter before (*D*_0_ (mm)) and after (*D*_1_ (mm)) thermal treatment [27] (Equation (1)):

(1) $$S\left(\%\right)=\frac{\left(D_{0}-D_{1}\right)}{D_{0}}\times 100$$

The mechanical resistance tests were carried out by the three points bending method (Lloyd Instrument, Bognor Regis, UK) to control the resistance of the membrane fired at different temperatures. The size of the samples is 45 × 12 × 2 mm^3^ and the distance separating the two points is 30 mm. This corresponds to the maximum bending stress at which the sample fractures.

The membrane samples were observed using a Merlin Scanning Electron Microscope (SEM) from Carl Zeiss (Baden-Württemberg, Germany) with an accelerating voltage of 5 kV. Elemental mapping was carried out using an energy-dispersive X-ray detector (EDX) SDD X-Max from Oxford Instruments (Abingdon, UK) operating at 10 kV. The preparation of our samples for SEM analyses consists of three main steps: (i) rinsing the surface with ethanol, (ii) drying the sample in an oven at 90 °C for 24 h (checking the stability of the sample weight), and (iii) applying a 4 nm conductive layer of palladium/platinum in a Cressington sputter coater 208HR.

The average pore size of the membrane was calculated using the extended Hagen–Poisseuille equation [28] (Equation (2)):

(2) $$d=2\sqrt{8\;J_{w}\;\delta \;\frac{\tau }{\varepsilon }\frac{\Delta X}{\Delta P}}$$

where *d* (m) is the pore diameter, *δ* (Pa. s) is the water viscosity, *J_w_* (m·s^−1^) is the water flux, ε (%) is the membrane porosity, *τ* is the tortuosity factor (2.5) for sphere particle packing, Δ*X* (m) is the membrane thickness, and Δ*P* (Pa) is the transmembrane pressure applied.

The chemical resistance was assessed by the mass loss of the membrane in HNO_3_ (0.2 M) solution and in NaOH (0.5 M) solution for a week [12].

### 2.4. Filtration Tests

**Figure 2:**
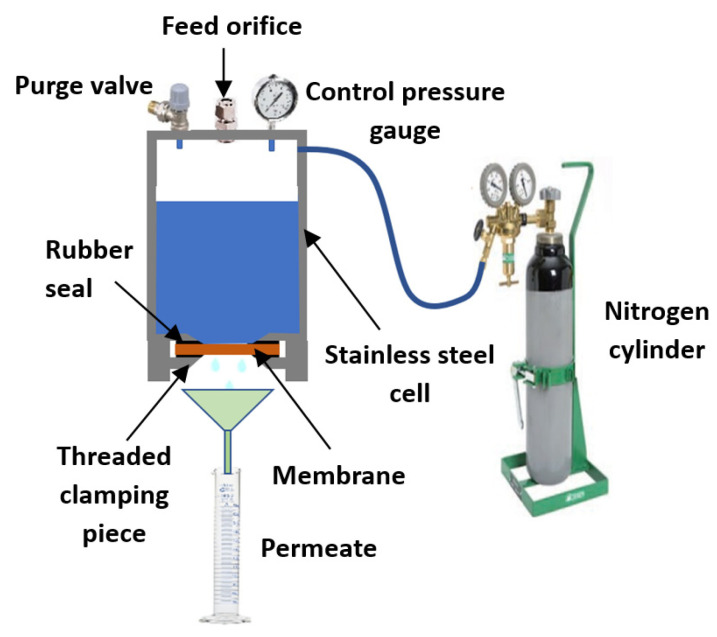
Schematic representation of the filtration system used (laboratory scale).

The performances of the composite membranes were evaluated at different transmembrane pressures (TMP) using a homemade set-up (Figure 2). A nitrogen gas cylinder was used to maintain the working pressure, which was controlled by a control pressure gauge and a purge valve. The membrane was placed between two rubber seals to seal the device (Figure 2). A threaded clamping piece was used to exert the pressure required to achieve this seal. The solution was introduced from the top via a filling orifice. Before the filtration tests, the synthesized membrane was soaked for 24 h in distilled water. Water permeability of the membrane was evaluated according to Darcy’s laws (Equations (3) and (4)):

(3) $$J_{w}=\frac{V}{At}$$

(4) $$J_{w}=L_{p}\times \Delta P$$

where *J_w_* (m·s^−1^) is the water flux, *V* (L) is the permeate volume collected during the time interval *t* (h), *A* is the effective membrane area (m^2^), *L_p_* is water permeability (L·h^−1^·bar^−1^·m^2^), and ∆*P* is the applied transmembrane pressure (bar).

The prepared membranes were used to treat the colored solution containing indigo blue (IB) dye. IB is an organic dye classified among the most important of the indigoid dyes. Its chemical formula is C_16_H_10_N_2_O_2_, and its chemical structure is given hereunder. IB is very useful in the textile industry, especially in the production of jeans. This dye is a dark blue crystalline powder that sublimes at 390–392 °C. It is insoluble in water, alcohol, or ether, but soluble in DMSO, chloroform, nitrobenzene, and concentrated sulfuric acid. IB must be reduced to a soluble form (leuco form) through a strong binding agent (sodium hydrosulfite in our case). In this study, the IB dye was provided by SITEX company located in Ksar Hellal–Tunisia and specialized in the textile industry.

The IB molecule absorbs light in the orange part of the spectrum (λ_max_ = 613 nm). It owes its deep color to the conjugation of the double bonds, i.e., the double bonds within the molecule are adjacent and the molecule is planar. In white indigo, the double bonds conjugation is blocked because the molecule is non-planar.
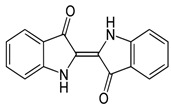


The physicochemical characterization of the raw and treated IB solution was determined by measuring three parameters: turbidity retention, COD, and color. The turbidity was measured by a turbidimeter (model 2100A, Hach, Loveland, CO, USA) in accordance with the standard method 2130B. The COD was measured using a colorimetric technique (COD 10119, Fisher Bioblock Scientific, Waltham, MA, USA). The color intensity was determined from absorbance values obtained with a UV/visible spectrophotometer (Aquanova Jenway) at the 620 nm wavelength.

The retention values were calculated using to the following equation (Equation (5)):

(5) $$R\left(\%\right)=\frac{\left(C_{f}-C_{p}\right)}{C_{f}}\times 100$$

where *C_f_* is the pollutant concentration in feed solution and *C_P_* is the pollutant concentration in permeate. The IB solution (feed stream) treated in this study had a turbidity of 30 NTU, a COD of 152.5 mg·L^−1^, and an absorbance of 0.061 at 620 nm.

### 2.5. Separation and Fouling Mechanisms

The IB retention by the membrane was evaluated using the mass balance equation (Equation (6)):

(6) $$V_{i}C_{i}=V_{p}C_{p}+V_{r}C_{r}$$

where *C_i_*, *C_p_*, and *C_r_* are the initial, permeate, and retentate IB concentrations, and *V_i_*, *V_p_*, and *V_r_* are, respectively, the initial, permeate, and retentate volumes.

To explain the permeate flux decrease during the MF operations, we have used the Hermia empirical model based on four equations related to (i) a complete pore-blocking (Equation (7)), (ii) a standard pore-blocking (Equation (8)), (iii) an intermediate pore-blocking (Equation (9)), and (iv) a cake filtration (Equation (10)). The details of this model are reported by Vinoth Kumar et al. [29].

(7) $$Ln\left(J_{w}^{-1}\right)=Ln\left(J_{w}^{-1}\right)+K_{1}t$$

(8) $$J_{w}^{-0.5}=J_{0}^{-0.5}+K_{2}t$$

(9) $$J_{w}^{-1}=J_{0}^{-1}+K_{3}t$$

(10) $$J_{w}^{-2}=J_{0}^{-2}+K_{4}t$$

where *J_w_* is the flux, *t* is the filtration time, *K* is the slope of the line, and *J*_0_ is the ordinate at the origin.

### 2.6. Determination of Fouling Resistances and Membrane Regeneration

The antifouling characteristics of our membranes were evaluated under an optimal pressure of 1 bar for 1 h by determining the flux recovery ratio (FRR) according to the following equation (Equation (11)):

(11) $$\mathrm{FRR}\left(\%\right)=\frac{Jw_{2}}{Jw_{1}}\times 100$$

*J_w_*_1_ is the water flux of the virgin membrane and *J_w_*_2_ is the water permeate flux of the fouled membrane. *J_w_*_2_ is determined after membrane rinsing with distilled water.

The membrane regeneration was accomplished initially by water rinsing followed by an acid–basic treatment with the circulation of 2 wt.% solutions of NaOH at 80 °C for 30 min, then a 2 wt.% solution of HNO_3_ at 60 °C also for 30 min. Finally, the membrane was rinsed with distilled water until a neutral pH was obtained [12]. The efficiency of the cleaning protocol was confirmed by measuring water permeability after the cleaning cycle, which must be almost equal to that of the new membrane. A water permeability reduction of 10% can be considered acceptable after some fifty uses and cycles of regeneration.

### 2.7. Germination Test

Germination experiments based on the AFNOR standard X31 201 were carried out to investigate the toxicity of the permeate and feed IB solution following the treatment utilizing the three prepared membranes. Twenty grains of linseed were used in glass Petri dishes with a cover and a layer of filter paper. The Petri dishes were then put in the dark for 10 days, into a controlled incubator at 20 °C, and the germination percentage (%) was calculated using the following Equation (12):

(12) $$GR\left(\%\right)=\frac{NGS}{NTS}\times 100\;$$

where *GR* is the germination percentage, *NGS* is the number of germinated seeds, and *NTS* is the total number of seeds.

## 3. Results and Discussion

### 3.1. Characterization of Flat Ceramic Membranes

#### 3.1.1. Visual Inspection of the Sintering Temperature

**Figure 3:**
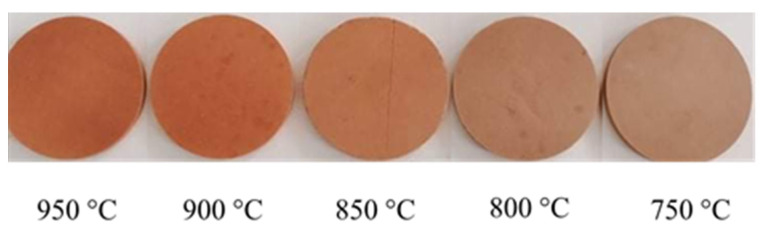
Visual aspect of composite membranes at different sintering temperatures.

Sintering is an essential stage in the production of ceramic membranes since it allows the development of appropriate mechanical strength; in addition, it governs the membrane pore size. Our membranes were examined at temperatures ranging from 750 to 950 °C to optimize the sintering temperature (Figure 3). It is observed from this figure that the color of the membrane changes from brown to dark brown when the sintering temperature increases. The more the firing temperature increases, the more the color of the membrane becomes darker. This color variation could be explained by the degree of iron oxidation [30]. In fact, when the oxidation temperature reached 850 °C or higher, the metallic luster disappeared, and the surface of the sample was covered with a layer of gray-black iron oxide. This external layer has no significant influence on the mechanical strength of the membrane. Therefore, the sintering temperature of these composite membranes must be higher than 850 °C.

At this temperature level (around 850–950 °C), the energy cost of membrane synthesis remains significantly lower than that required for other raw materials, such as titanium or alumina oxides. The sintering temperature for the latter two materials exceeds 1200 °C. This energetic aspect reinforces the low-cost character of the membranes prepared in this study.

#### 3.1.2. Shrinkage

**Figure 4:**
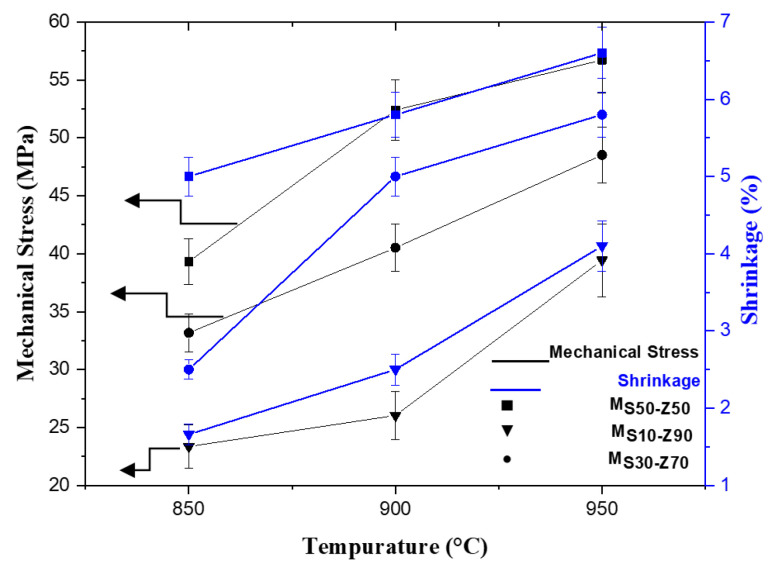
Variation of mechanical strength and membrane shrinkage with the sintering temperature and composition.

Generally, shrinkage is related to particle rearrangement or/and weight loss caused by evaporation, combustion, or decomposition during thermal treatment [31]. The shrinkage of fabricated composite membranes M_S10-Z90_, M_S30-Z70_, and M_S50-Z50_ as a function of the sintering temperature (from 850 to 950 °C) is shown in Figure 4. The shrinkage increased from 1.66 to 7.00 wt.% with the increase of the added amount of smectite (from 10 to 50 wt.%) as well as with the sintering temperature (from 850 to 950 °C) due to the densification phenomenon. It should be noted that a similar occurrence was discovered in the literature using natural phosphate [32], natural pozzolan, and micronized phosphate [33], whereby the shrinkage ranges of 3–5 wt.% at the firing temperature varied between 900 °C and 950 °C.

#### 3.1.3. Mechanical Strength

Mechanical resistance evolution of the different composite membranes, M_S10-Z90_, M_S30-Z70_, and M_S50-Z50_ with sintering temperatures is shown in Figure 4. The mechanical strength increases when the sintering temperature increases from 850 °C to 950 °C and the smectite ratio increases from 10 wt.% to 50 wt.%. These results are attributed to the sintering degree as well as to the densification of materials, which leads to a more consolidated ceramic body at a higher temperature and also to the smectite behavior [27,33,34,35]. Therefore, for energetic consideration, 850 °C can be chosen as the optimum sintering temperature due to the low shrinkage observed at this temperature not exceeding 5% and high mechanical strengths exceeding 23 MPa.

#### 3.1.4. Morphology and Pore Size Analysis

**Figure 5:**
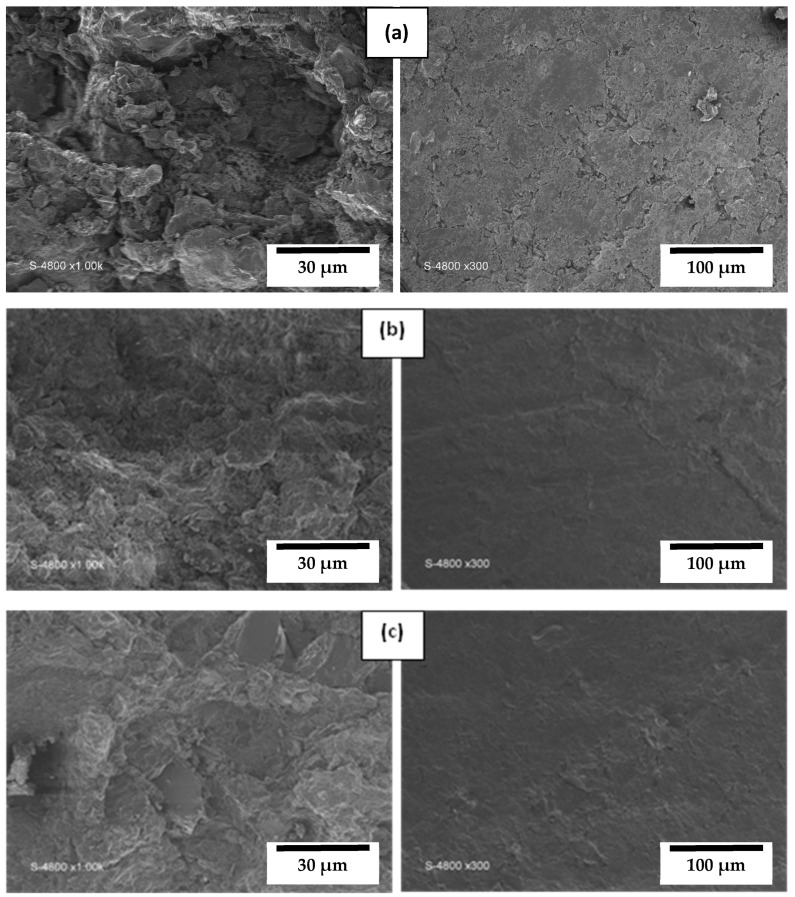
SEM micrographs of M_S10-Z90_ (**a**), M_S30-Z70_ (**b**), and M_S50-Z50_ (**c**) sintered at 850 °C.

In order to evaluate the microstructure, SEM was used to examine the top surface of manufactured M_S10-Z90_, M_S30-Z70_, and M_S50-Z50_ membranes (Figure 5a–c) sintered at 850 °C. SEM micrographs show that all membrane surfaces were homogenous and without cracks. The addition of smectite had a significant effect on membrane morphology. The densification of the surfaces occurred with pore closure. It is clear that the more smectite is incorporated into the zeolite membrane, the more the intergranular pores become smaller. This finding proves that incorporating smaller particles (S) between zeolite grains can reduce the membrane pore diameter [36,37]. At the same time, increasing the smectite content increases membrane consolidation because zeolite must be sintered at higher temperatures.

EDX analysis of smectite/zeolite membranes sintered at 850 °C indicated important signals of Si, Al, O, Ca, Mg, Na, and K, which are the most characteristic elements of zeolite and smectite. In addition, P and S peaks are related to smectite composition. Small carbon-related peaks were detected, which may be due to impurities [38].

The average pore size of the different membranes was estimated using the Hagen–Poiseuille equation. Its value is 0.98 µm, 0.95 µm, and 0.75 µm for M_S10-Z90_, M_S30-Z70_, and M_S50-Z50_, respectively. This result indicates that the new composite membranes are classified as macro-porous and can be good candidates to be functional in the microfiltration domain.

#### 3.1.5. Chemical Resistance

**Figure 6:**
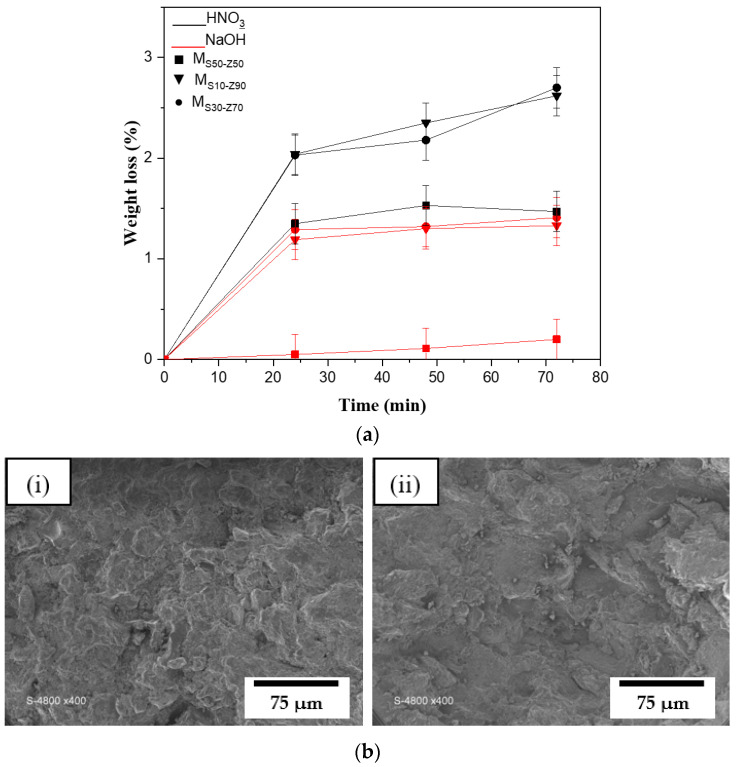
(**a**) Weight loss of different membranes sintered at 850 °C in acidic and basic solutions: M_S10-Z90_, MS_30-Z70_, and M_S50-Z50_. (**b**) SEM photos of M_Z90-S10_ (**i**) before and (**ii**) after chemical treatment.

Membrane separation procedures, particularly in MF, require disinfection and cleaning operations. The chemical resistance of ceramic membranes is typically tested at severe pH levels to ensure their viability [36]. The M_S10-Z90_, M_S30-Z70_, and M_S50-Z50_ membranes sintered at 850 °C were subjected to chemical resistance tests for 72 h at room temperature. Figure 6a shows the variation of membranes’ weight loss over time. In fact, the weight loss was negligible when M_S50-Z50_ was placed into a soda-aqueous solution for 72 h, and for the two other membranes (M_S10-Z90_ and M_S30-Z70_), it did not exceed 1.5 wt.%. However, in an acid solution with the same conditions in terms of time and temperature, the weight loss remains low for the M_S50-Z50_ but quickly reaches 2–3 wt.% for the two other membranes. The lesser chemical resistance of membranes in an acid environment is mainly due to the high solubility of smectite at low pH solutions [39]. In spite of this mass loss, which remains very acceptable, and in view of the long treatment time (72 h) and the low cost of our membranes, the industrial balance remains very much in favor of these membranes.

The comparison between the SEM photos (Figure 6b) for the retained membrane M_Z90-S10_ before and after chemical treatment (acidic treatment) revealed that no change in the membrane morphology was observed.

After chemical resistance tests, no phenomenon was observed in terms of color change, degradation, and aging. As a result, the developed membranes have good chemical resistance and are adapted to applications involving acid and base media. These results corroborate the findings from previous studies [27,40,41].

#### 3.1.6. Water Permeability

**Figure 7:**
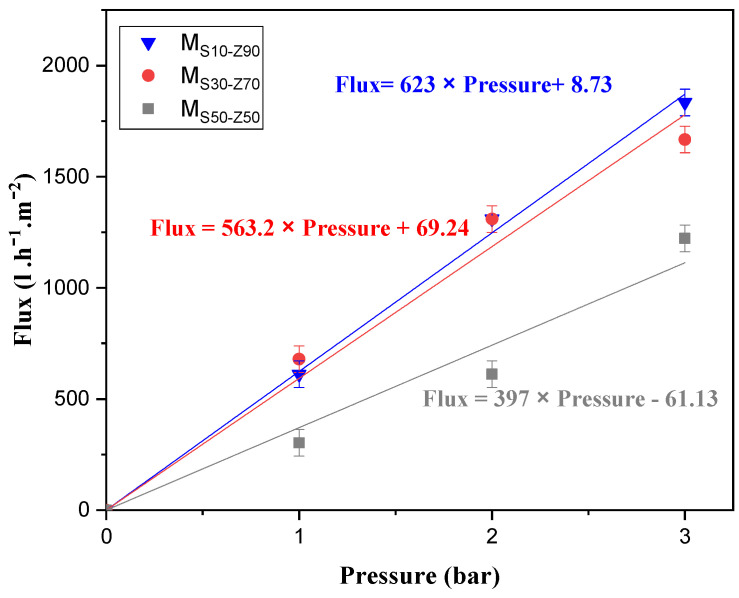
Determination of water permeability for the different membranes sintered at 850 °C: M_S10-Z90_, M_S30-Z70_ and M_S50-Z50_.

The water permeability of the composite membranes, sintered at 850 °C as a function of transmembrane pressure, is illustrated in Figure 7. It can be seen that water permeability decreases from 623 L·h^−1^·m^−2^·bar^−1^ to 371 L·h^−1^·m^−2^·bar^−1^ when the smectite ratio increases from 10 wt.% to 50 wt.%. This can be explained by the decrease in the number of pores due to surface densification. This observation is compatible with a less porous aspect already observed in SEM micrographs. These permeability values were suitable, confirming thereby that these membranes operate in the microfiltration domain.

### 3.2. Removal of Indigo Blue Dye

#### 3.2.1. Treatment of Indigo Blue Wastewater by Composite MF Membranes

**Figure 8:**
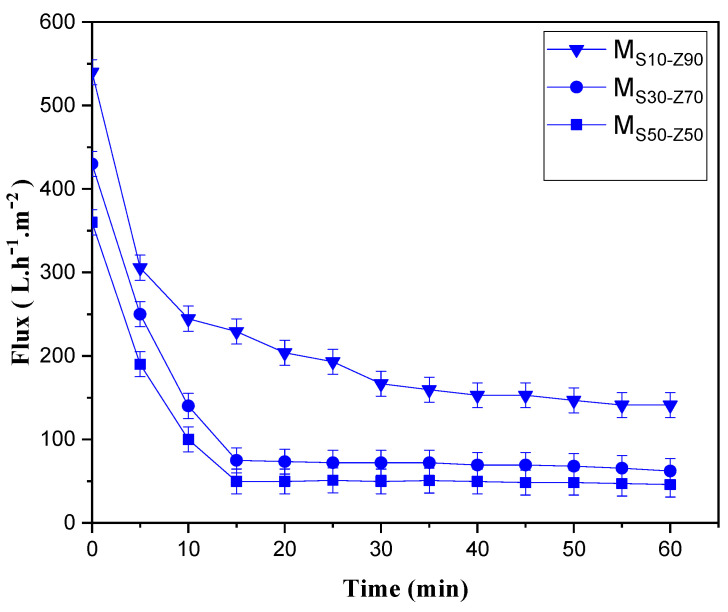
Evolution of permeate flux with time for M_S10-Z90_, M_S30-Z70_, and M_S50-Z50_ membranes sintered at 850 °C.

The treatment of industrial wastewater can be achieved by the membrane process. In the current study, the MF membrane was applied to the treatment of synthetic effluent using IB dye. Membrane filtration experiments were carried out at room temperature and a transmembrane pressure of 1.0 bar using the three prepared membranes M_S10-Z90_, M_S30-Z70_, and M_S50-Z50_ sintered at 850 °C. For the M_S10-Z90_ membrane, the permeate flux decreased progressively during the first 30 min from 360 to 170 L·h^−1^·m^−2^ and then stabilized at this value. This reduction in flux is explained by the formation of concentration polarization and fouling caused by the interaction between the solution and the membrane material [42]. Nevertheless, for M_S30-Z70_ and M_S50-Z50_ membranes, a quick and significant flux reduction was observed during the first 15 min of filtration. The stabilized permeate flux was established at 70 L·h^−1^·m^−2^ and 50 L·h^−1^·m^−2^, respectively (Figure 8). These obtained values of stabilized permeate flux were in compliance with the average pore size values of 0.98 µm, 0.95 µm, and 0.75 µm for M_S10-Z90_, M_S30-Z70_, and M_S50-Z50_, respectively.

**Figure 9:**
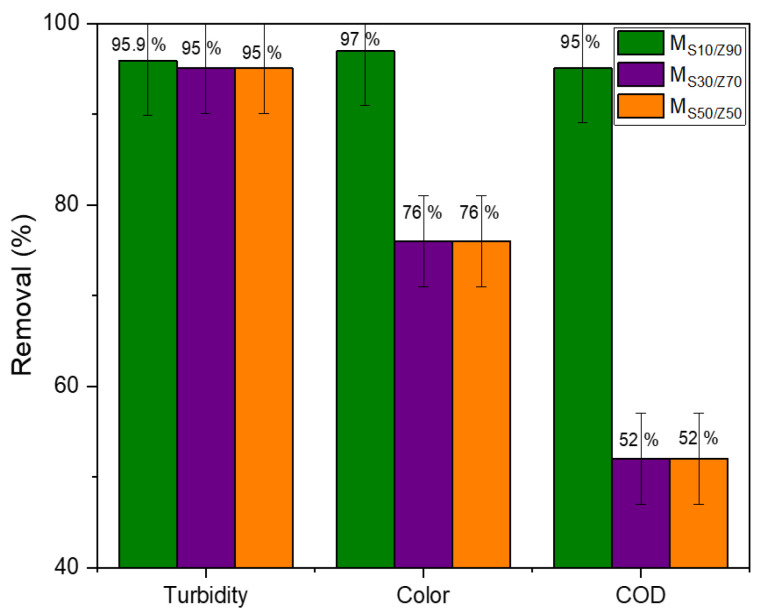
Evolution of the retention of various pollutants (turbidity, COD, color (Abs._λmax_)) for the different synthesized membranes.

The removal of contaminants (turbidity, COD, and color) at 1 bar was determined (Figure 9). For all membranes, an almost total turbidity removal was observed exceeding 90%, while a very good retention of color and COD was enrolled for M_S10-Z90_ of 95%, and only 76% and 52% were observed, respectively, for M_S30-Z70_ and M_S50-Z50_ sintered at 850 °C. Based on the literature, a low-cost flat ceramic membrane exhibits almost total turbidity retention and good COD removal superior to 67% [13,14,15,27,32,36].

#### 3.2.2. Separation Mechanisms and Fouling Study

The variation of the permeate flux with time gives an estimation of the importance of membrane fouling illustrated by the reduction of permeate flux with time due to the deposition of foulants onto the membrane surface and into the pores. Figure 8 shows the permeate flux profile for the three elaborated membranes. Two behaviors were observed. For M_S10-Z90_, the permeate flux decreased progressively at the beginning of the filtration process and stabilized at 170 L·h^−1^ m^−2^ after 30 min. However, a drastic decrease of the permeate flux was observed for the two other synthesized membranes, M_S30-Z70_ and M_S50-Z50_, which stabilized rapidly after 15 min at 70 L·h^−1^·m^−2^ and 50 L·h^−1^·m^−2^, respectively.

Table 3 shows that the mass balance between the membrane inlet and outlet during filtration was almost satisfactory for both M_S30-Z70_ and M_S50-Z50_ membranes (an error of less than 1%); however, the error is higher than 4% for M_S10-Z90_. The difference is explained by the dye adsorption at the surface of the membrane, which is fostered by the increase of the zeolite percentage in the membrane composition. After the saturation process, the permeate flux continues to decrease more slightly due to the accumulation of dye molecules on the surface and at the entrance of the pores. Other works in the literature observed similar behavior and attributed it to membrane pore-blocking [15]. As a result, the layer formed on the surface of the membrane increases in thickness as filtration progresses and the effective diameters of the membrane pores decrease, which explains the high retention of dye molecules.

Table 4 presents associated characteristics for four pore-blocking models in terms of slope, y-intercept, and R^2^. It is evident that the model describing experimental data with the best R^2^ value (almost 1) is considered a suitable model that refers to the most appropriate fouling mechanism.

These results suggest that the reduction of M_S10-Z90_ flux can be predicted by cake filtration, proving the zeolite adsorption properties already demonstrated. The permeate flux reduction in the case of M_S30-Z70_ can be explained by standard pore-blocking, which suggests that the membrane contains particles smaller than or equal to the membrane pores. For the M_S50-Z50_ membrane, R^2^ is relatively low (<0.900); therefore, it can be deduced that the Hermia model did not correlate with the experimental data. The suggested pore-blocking model for M_S10-Z90_ and the retained result for M_S50-Z50_ were similar to the Kaolin membranes prepared in our laboratory [38]. Additionally, Beqqour et al. [33] proved standard pore-blocking using a flat membrane made from natural pozzolan and micronized phosphate like our M_S30-Z70_ membrane.

The flux recovery ratio (FRRs) for each of the three synthesized membranes were found to be 40.1%, 30.2%, and 39.1% for M_S10-Z90_, M_S30-Z70_, and M_S50-Z50_, respectively. The FRR values for the different membranes are in accordance with the results found previously from the Hermia model. For the M_S10-Z90_ membrane, the FRR seems to be the highest value due to the use of a substantial amount of zeolite (90%), which favors adsorption, while the lowest value (FRR = 30.2%) obtained with the M_S30-Z70_ membrane is attributed to adsorption and pore-blocking.

It can be stated that the M_S10-Z90_ membrane exhibited a higher removal of color and COD (>95%), with an excellent permeate flux of 170 L·h^−1^·m^−2^ compared to M_S30-Z70_ and M_S50-Z50_ membranes. In addition, the permeate flux of the developed membrane M_S10-Z90_ recovered to 40% of the initial flux (the higher FRR = 40.13%). Therefore, the mixture of 10 wt.% of smectite with 90 wt.% of zeolite was chosen as an optimal composition for the preparation of the microfiltration composite membrane. The determination of the fouling properties show that chemical cleaning is necessary to restore the initial membrane performances for the textile wastewater treatment due to an intensive membrane fouling [29].

## 4. Germination Test

**Figure 10:**
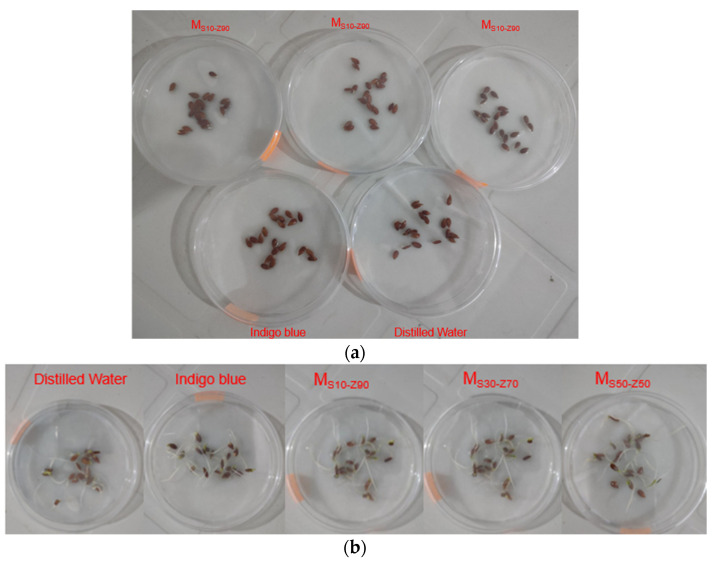
(**a**) Results of the germination tests using M_S10-Z90_, M_S30-Z70_, and M_S50-Z50_ after the first day. (**b**) Results of the germination tests using M_S10-Z90_, M_S30-Z70_, and M_S50-Z50_ after 10 days.

Figure 10a,b depicts the results of the germination tests using linseed grains treated by the same volume of several solutions: untreated IB solution before treatment (25 mg·L^−1^) and three solutions of IB after treatment by different composite membranes and distilled water.

The inhibiting effect caused by the toxicity of the solution has a direct impact on the number of germinated grains. The germination in the petri dish containing the distilled water was expected to be normal after 10 days, reaching 100%; however, the germination rate was lower and did not exceed 80%when using the raw IB solution, which proves that the toxicity of this solution prevents the germination of linseed grains. In addition, the germination in the treated IB solution using M_S10-Z90_, M_S30-Z70_, and M_S50-Z50_ membranes demonstrates great effectiveness of the synthesized membranes for the removal of solution toxicity by the microfiltration process, as the removal rate reached 100%, 95%, and 85% for M_S10-Z90_, M_S30-Z70_, and M_S50-Z50_, respectively (Table 5), on the final day. From these results, it is clear that the total germination of grains treated by the permeate of M_S10-Z90_ membrane is identical to that treated with distilled water, which proves again that the mixture of 10% smectite and 90% zeolite is the best composition.

## 5. Comparative Study

The performances of the three synthesized composite membranes were compared with the different types of low-cost membranes reported in the literature. The membrane characteristics obtained by the dry pressing process applied to the treatment of dyeing wastewater are illustrated in Table 6. It is clear that M_S10-Z90_ composite MF membranes based on smectite and zeolite present a lower sintering temperature of 850 °C compared with composite membranes from low-cost materials sintered in the range of 900–1100 °C [29,31,32,33,35,42]. In addition, M_S10-Z90_, M_S30-Z70_, and M_S50-Z50_ membranes displayed good properties, especially in terms of their mechanical resistance, which is higher than 23 MPa. From the literature studies, mechanical strength in the range of 14.42–16.13 MPa was observed for three flat membranes from Moroccan clays [30]. On the other hand, in the works of Manni et al. [43], the synthesized membrane made from natural magnesite showed a very low mechanical resistance of only 6.1 MPa despite being sintered at a relatively high sintering temperature of 1100 °C. Taking into account the filtration performances, the value of permeate flux (70–170 L·h^−1^·m^−2^ at 1 bar) found in this study is higher than the value mentioned by Beqqour et al. [33] (20 L·h^−1^·m^−2^ at 0.12 bar) using a new flat membrane made from natural pozzolan and micronized phosphate. Also, different MF flat membranes based on phosphate, clay, and waste materials reported in the literature presented permeate flux in the range of 30–45 L·h^−1^·m^−2^, which is lower than the values achieved in this work [27,32,36].

It is clear that the MF membranes developed in this study present good performance compared with other membranes reported in the literature.

This comparative study indicates that M_S10-Z90_ displays a great potential for the application in textile industry wastewater treatment.

## 6. Conclusions

Low-cost microfiltration ceramic composite membranes were successfully prepared using a mixture of natural zeolite and smectite at different percentages. The effect of the sintering temperature (850–950 °C) demonstrated that the increase in the temperature remarkably improved the mechanical strength and shrinkage. On the other hand, the smectite addition (10–50 wt.%) significantly reduced the pore size and water permeate flux of the composite membranes. Furthermore, it was found that the best membrane M_S10-Z90_ (10 wt.% smectite and 90 wt.% zeolite) sintered at 850 °C had an average pore size of 0.98 μm, water permeability of 623 L·h^−1^·m^−2^·bar^−1^, and mechanical strength of 23 MPa. M_S10-Z90_ was successfully used for the elimination of IB dye, with a removal rate up to 95% of color and COD as well as an excellent stabilized permeate flux of 170 L·h^−1^·m^−2^ and a good membrane regeneration after use. The highest FRR value exceeding 40% was also determined in the case of M_S10-Z90_.

Germination tests indicated that the MF permeate can be reused for irrigation in a circular economy strategy. Finally, M_S10-Z90_ has the dual advantage of consuming low amounts of energy and using abundant and inexpensive raw materials.

## Figures and Tables

**Table 1:** Composition of smectite and zeolite powders.

Composition	LOI *	SiO_2_	TiO_2_	Al_2_O_3_	Fe_2_O_3_	CaO	CaCO_3_	MgO	ZnO	K_2_O	SO_3_	Na_2_O	P_2_O_5_
Smectite (wt.%)	16.33	49.90	---	19.77	6.49	1.01	---	1.61	---	1.57	0.76	0.81	0.44
Zeolite (wt.%)	---	73.3	0.03	11.75	1.53	3.79	6.8	1.19	0.01	1.44	---	0.36	---

* LOI: loss on ignition.

**Table 2:** Composition of the prepared membranes.

Membrane	Smectite (wt.%)	Zeolite (wt.%)
M_S10-Z90_	10	90
M_S30-Z70_	30	70
M_S50-Z50_	50	50

**Table 3:** Determination of mass balance for color removal using MF composite membranes (ΔP = 1 bar, *C_i_* = 27.21 mg·L^−1^, *V_i_* = 2 L, *t* = 1 h).

	*C_i_V_i_*	*C_p_V_p_* + *C_r_V_r_*	Error (%)
M_S10-Z90_	54.4	52.1	4.20
M_S30-Z70_	54.4	54.0	0.63
M_S50-Z50_	54.4	54.1	0.60

**Table 4:** Parameters related to different pore-blocking models and flux recovery.

Blocking Model	M_S10-Z90_			M_S30-Z70_			M_S50-Z50_		
	*K*	*J*_0_	R^2^	*K*	*J*_0_	R^2^	*K*	*J*_0_	R^2^
Complete pore-blocking	1.04 × 10^−2^	246.3	0.89	3.6 × 10^−3^	79.3	0.93	1.7 × 10^−3^	51.94	0.72
Standard pore-blocking	4 × 10^−4^	252.7	0.91	2 × 10^−4^	79.6	0.94	1 × 10^−4^	52.00	0.72
Intermediate pore-blocking	6 × 10^−5^	263.2	0.92	5 × 10^−5^	80.0	0.93	4 × 10^−5^	50.54	0.72
Cake filtration	7 × 10^−7^	316.2	0.95	2 × 10^−6^	70.7	0.91	1 × 10^−6^	50.00	0.72

**Table 5:** Different percentages of the germination test.

Samples	IB	Distilled Water	M_S10-Z90_	M_S30-Z70_	M_S50-Z50_
Percentage of germination (%)	80	100	100	95	85

**Table 6:** Comparison of the properties and performances of composite flat membranes developed in the present work with those reported in the literature.

Raw Materials	Sintering T (°C)	Pore Size (μm)	Mechanical Strength (MPa)	Permeate Flux (L·h^−1^·m^−2^)	COD * (%)	Reference
Pozzolan + phosphate	1050	1.33	15.6	20	-	[33]
phosphate/kaolinite	1000	0.35	40.2	40	74	[32]
Clay of Meknes (CM)	950	1.80	14.80	90	-	[30]
Clay of Fez (FCF)		1.5	16.13	50	-	
Granular clay of Fez (GCF)		2.84	14.42	30	-	
Natural magnesite	1100	1.12	6.1	61	69.7	[43]
Sludge + natural clay	1050	0.92	14.5	35	67.9	[27]
Bentonite	950	1.70	22.00	40	99	[36]
M_S10-Z90_	850	0.983	23.36	170	95.08	This Work
M_S30-Z70_		0.958	33.10	70	78.68	
M_S50-Z50_		0.750	39.44	50	52.45	

* COD (%): The percentage removal of chemical demand oxygen.

## Data Availability

The data presented in this study are available on request from the corresponding author.

## References

[1] Reddy C.V., Reddy I.N., Akkinepally B., Harish V.V.N., Reddy K.R., Jaesool S. (2019). Mn-Doped ZrO_2_ Nanoparticles Prepared by a Template-Free Method for Electrochemical Energy Storage and Abatement of Dye Degradation. Ceram. Int..

[2] Rani S.L.S., Kumar R.V. (2021). Insights on Applications of Low-Cost Ceramic Membranes in Wastewater Treatment: A Mini-Review. Case Stud. Chem. Environ. Eng..

[3] Liu H., Guo W., Li Y., He S., He C. (2018). Photocatalytic Degradation of Sixteen Organic Dyes by TiO_2_/WO_3_-Coated Magnetic Nanoparticles under Simulated Visible Light and Solar Light. J. Environ. Chem. Eng..

[4] Wang X., Jiang J., Gao W. (2022). Reviewing textile wastewater produced by industries: Characteristics, environmental impacts, and treatment strategies. Water Sci. Technol..

[5] Siwińska-Ciesielczyk K., Świgoń D., Rychtowski P., Moszyński D., Zgoła-Grześkowiak A., Jesionowski T. (2020). The Performance of Multicomponent Oxide Systems Based on TiO_2_, ZrO_2_ and SiO_2_ in the Photocatalytic Degradation of Rhodamine B: Mechanism and Kinetic Studies. Colloids Surf. Physicochemical. Eng. Asp..

[6] Fu Y., Viraraghavan T. (2001). Fungal Decolorization of Dye Wastewaters: A Review. Bioresour. Technol..

[7] Fahoul Y., Tanji K., Zouheir M., Mrabet I.E., Naciri Y., Hsini A., Nahali L., Kherbeche A.A. (2022). Novel River Sediment@ZnOCo Nanocomposite for Photocatalytic Degradation and COD Reduction of Crystal Violet under Visible Light. J. Mol. Struct..

[8] Omwene P.I., Can O.T., Öz U.M., Keyikoğlu R. (2023). Investigating the removal efficiency of different textile dye classes from wastewater by electrocoagulation using aluminum electrode. Int. J. Environ. Sci. Technol..

[9] Maeen M.d., Akter K., Nasrin Haq U., Islam M., Abbas Uddin M. (2022). Textile-apparel manufacturing and material waste management in the circular economy: A conceptual model to achieve sustainable development goal (SDG) 12 for Bangladesh. Clean. Environ. Syst..

[10] Khatooni H., Peighambardoust S.J., Foroutan R., Mohammadi R., Ramavandi B. (2023). Adsorption of methylene blue using sodium carboxymethyl cellulose-g-poly (acrylamide-co-methacrylic acid)/Cloisite 30B nanocomposite hydrogel. J. Polym. Environ..

[11] Jarvis P., Carra I., Jafari M., Judd S.J. (2022). Ceramic vs polymeric membrane implementation for potable water treatment. Water Res..

[12] Aloulou W., Aloulou H., Jadda A., Chakraborty S., Amar B.R. (2020). Characterization of an Asymmetric Ultrafiltration Membrane Prepared from TiO2-Smectite Nanocomposites Doped with Commercial TiO_2_ and Its Application to the Treatment of Textile Wastewater. Euro-Mediterr. J. Environ. Integr..

[13] Achiou B., Elomari H., Ouammou M., Albizane A.A., Bennazha J.S., Younssi A., El Hassani I.-E., Aaddane A. (2016). Elaboration and Characterization of Flat Ceramic Microfiltration Membrane Made from Natural Moroccan Pozzolan (Central Middle Atlas). J. Mater. Environ. Sci..

[14] Mouiya M., Abourriche A., Bouazizi A., Benhammou A., El Hafiane Y., Abouliatim Y., Nibou L., Oumam M., Ouammou M., Smith A. (2018). Flat ceramic microfiltration membrane based on natural clay and Moroccan phosphate for desalination and industrial wastewater treatment. Desalination.

[15] Samhari O., Younssi S.A., Rabiller-Baudry M., Loulergue P., Bouhria M., Achiou B., Ouammou M. (2020). Fabrication of Flat Ceramic Microfiltration Membrane from Natural Kaolinite for Seawater Pretreatment for Desalination and Wastewater Clarification. Desalin. Water Treat..

[16] Emani S., Uppaluri R., Purkait M.K. (2013). Preparation and characterization of low-cost ceramic membranes for mosambi juice clarification. Desalination.

[17] Majouli A., Tahiri S., Younssi S.A., Loukili H., Albizane A. (2012). Elaboration of new tubular ceramic membrane from local Moroccan Perlite for microfiltration process. Application to treatment of industrial wastewaters. Ceram. Int..

[18] Mezquita A., Monfort E., Ferrer S., Gabaldón-Estevan D. (2017). How to reduce energy and water consumption in the preparation of raw materials for ceramic tile manufacturing: Dry versus wet route. J. Clean. Prod..

[19] Mosadeghkhah A., Alaee M.A., Mohammadi T. (2007). Effect of sintering temperature and dwell time and pressing pressure on Ba_0.5_Sr_0.5_Co_0.8_Fe_0.2_O_3−δ_ perovskite-type membranes. Mater. Des..

[20] Lorente-Ayza M.M., Mestre S., Menéndez M., Sánchez E. (2015). Comparison of extruded and pressed low-cost ceramic supports for microfiltration membranes. J. Eur. Ceram. Soc..

[21] Lagdali S., Miyah Y., El-Habacha M., Mahmoudy G., Benjelloun M., Iaich S., Zerbet M., Chiban M., Sinan F. (2023). Performance assessment of a phengite clay-based flat membrane for microfiltration of real-wastewater from clothes washing: Characterization, cost estimation, and regeneration. Case Stud. Chem. Environ. Eng..

[22] Hussain I., Iqbal M., Nawaz M., Rasheed R., Perveen A., Mahmood S., Yasmeen A., Wahid A. (2013). Effect of sugar mill effluent on growth and Antioxidative Potential of Maize Seedling. Int. J. Agric. Biol..

[23] Kathirval P. (2011). The effect of dye effluent on growth, yield and biochemical attributes of Bengal Gram (*Cicer arietinum* L.). Int. J. Appl. Biol. Pharm..

[24] Mahawar P., Akhtar A. (2019). Impact of Dye Effluent on Seed Germination, Seedling Growth and Chlorophyll Content of Soybean (*Glycine max* L.). Int. J. Sci. Res..

[25] Aloulou W., Hamza W., Aloulou H., Oun A., Khemakhem S., Jada A., Chakraborty S., Curcio S., Amar R.B. (2018). Developing of Titania-Smectite Nanocomposites UF Membrane over Zeolite Based Ceramic Support. Appl. Clay Sci..

[26] Aloulou H., Bouhamed H., Ghorbel A., Ben Amar R., Khemakhem S. (2017). Elaboration and Characterization of Ceramic Microfiltration Membranes from Natural Zeolite: Application to the Treatment of Cuttlefish Effluents. Desalination Water Treat..

[27] Mouratib R., Achiou B., Krati M.E., Younssi S.A., Tahiri S. (2020). Low-cost ceramic membrane made from alumina- and silica-rich water treatment sludge and its application to wastewater filtration. J. Eur. Ceram. Soc..

[28] Kaur H., Bulasara V.K., Gupta R.K. (2016). Effect of carbonates composition on the permeation characteristics of low-cost ceramic membrane supports. J. Ind. Eng. Chem..

[29] Kumar R.V., Ghoshal A.K., Pugazhenthi G. (2015). Elaboration of novel tubular ceramic membrane from inexpensive raw materials by extrusion method and its performance in microfiltration of synthetic oily wastewater treatment. J. Membr. Sci..

[30] Elomari H., Achiou B., Ouammou M., Albizane A., Bennazha J., Younssi S.A., Elamrani I. (2016). Elaboration and characterization of flat membrane supports from Moroccan clays. Application for the treatment of wastewater. Desalination Water Treat..

[31] Tansel B., Bao W.Y., Tansel I.N. (2000). Characterization of fouling kinetics in ultrafiltration systems by resistances in series model. Desalination.

[32] Belgada A., Achiou B., Younssi S.A., Charik F.Z., Ouammou M., Jason C.A., Benhida R., Khaless K. (2021). Low-cost ceramic microfiltration membrane made from natural phosphate for pretreatment of raw seawater for desalination. J. Eur. Ceram. Soc..

[33] Beqqour D., Achiou B., Bouazizi A., Ouaddari H., Elomari H., Ouammou M., Bennazha J., Younssi S.A. (2019). Enhancement of microfiltration performances of pozzolan membrane by incorporation of micronized phosphate and its application for industrial wastewater treatment. J. Environ. Chem. Eng..

[34] Suresh K., Katara N. (2021). Design and Development of Circular Ceramic Membrane for Wastewater Treatment. Mater. Today Proc..

[35] Saja S., Bouazizi A., Achiou B., Ouammou M., Albizane A., Bennazha J., Younssi S.A. (2018). Elaboration and Characterization of Low-Cost Ceramic Membrane Made from Natural Moroccan Perlite for Treatment of Industrial Wastewater. J. Environ. Chem. Eng..

[36] Bouazizi A., Saja S., Achiou B., Ouammou M., Calvo J.I., Aaddane A., Younssi S.A. (2016). Elaboration and characterization of a new flat ceramic MF membrane made from natural Moroccan bentonite. Appl. Treat. Ind. Wastewater Appl. Clay Sci..

[37] Bousbih S., Errais E., Darragi F., Duplay J., Trabelsi-Ayadi M., Daramola M.O., Amar R.B. (2021). Treatment of textile wastewater using monolayered ultrafiltation ceramic membrane fabricated from natural kaolin clay. Environ. Technol..

[38] Aloulou H., Aloulou W., Duplay J., Baklouti L., Dammak L., Amar B.R. (2022). Development of Ultrafiltration Kaolin Membranes over Sand and Zeolite Supports for the Treatment of Electroplating Wastewater. Membranes.

[39] Masmoudi S., Larbot A., Feki E.H., Amar R.B. (2007). Elaboration and Characterization of Apatite Based Mineral Supports for Microfiltration and Ultrafiltration Membranes. Ceram. Int..

[40] Mestre S., Gozalbo A., Lorente-Ayza M.M., Sánchez E. (2019). Low-Cost Ceramic Membranes: A Research Opportunity for Industrial Application. J. Eur. Ceram. Soc..

[41] Hubadillah S.K., Othman M.H.D., Matsuura T., Ismail A.F., Rahman M.A., Harun Z., Jaafar J., Nomura M. (2018). Fabrications and applications of low-cost ceramic membrane from kaolin: A comprehensive review. Ceram. Int..

[42] Rad L.R., Anbia M.M. (2021). Zeolite-Based Composites for the Adsorption of Toxic Matters from Water: A Review. J. Environ. Chem. Eng..

[43] Manni A., Achiou B., Karim A., Harrati A., Sadik C., Ouammou M., Younssi S.A., Bouari A. (2020). New low-cost ceramic microfiltration membrane made from natural magnesite for industrial wastewater treatment. J. Environ. Chem. Eng..

